# White matter dissection with the Klingler technique: a literature review

**DOI:** 10.1007/s00429-020-02157-9

**Published:** 2020-11-09

**Authors:** Tomasz A. Dziedzic, Artur Balasa, Mateusz P. Jeżewski, Łukasz Michałowski, Andrzej Marchel

**Affiliations:** 1 Department of Neurosurgery, Medical University of Warsaw, Warsaw, Poland; 2 Department of Pathology, Medical University of Warsaw, Warsaw, Poland

**Keywords:** Anatomy, White matter, Fiber microdissection, Klingler technique, Tractography

## Abstract

The aim of this literature review is to present a summary of the published literature relating the details of the different modifications of specimen preparation for white matter dissection with the Klingler technique. For this review, 3 independent investigators performed an electronic literature search that was carried out in the Pubmed, Scopus and Web of Science databses up to December 2019. Furthermore, we performed citation tracking for the articles missed in the initial search. Studies were eligible for inclusion when they reported details of at least the first 2 main steps of Klingler’s technique: fixation and freezing. A total of 37 full-text articles were included in the analysis. We included original anatomical studies in which human white matter dissection was performed for study purposes. The main three steps of preparation are the same in each laboratory, but the details of each vary between studies. Ten percent formalin is the most commonly used (34 studies) solution for fixation. The freezing time varied between 8 h and a month, and the temperature varied from − 5 to − 80 °C. After thawing and during dissections, the specimens were most often kept in formalin solution (13), and the concentration varied from 4 to 10%. Klingler’s preparation technique involves three main steps: fixation, freezing and thawing. Even though the details of the technique are different in most of the studies, all provide subjectively good quality specimens for anatomical dissections and studies.

## Introduction

The white matter of the brain is composed of myelinated nerve axons. These axons, when sharing a common origin and destination, form compact structures such as white matter fascicules or tracts. The orientation of axons within the brain can be traced noninvasively through diffusion MRI (dMRI). This technique provides the possibility to study the anatomy of white matter and connections between different cortical regions. Specific fascicules can be isolated from the whole brain based on the anatomical knowledge that comes from classical anatomical white matter dissections. The identification of potentially new tracts based on dMRI findings has also been proven with anatomical dissection. The most common anatomical white matter dissection techniques, currently, are modifications of the initial technique described by (Klingler 1935). According to the initial technical note, the preparation method consists of a systematic approach including three main steps. In the first step, the brain is removed from the body and fixed; in the second step, one specimen is frozen; and in the third step, the specimen is thawed and, finally, white matter tracts are dissected. Klingler performed his dissections mainly with homemade wooden spatulas without any magnification. It is possible to dissect the main white matter tracts within the cerebral hemisphere with the naked eye, but for smaller tracts, such as those within the brain stem, microscopes and microsurgical tools are of great importance.

When following the methods section of manuscripts that include white matter dissection, most authors state that their technique is following Klingler’s original technique (Capilla-Guasch et al. 2019; De Benedictis et al. 2014; Di Carlo et al. 2019; Dini et al. 2013; Fernandez-Miranda et al. 2008; Flores-Justa et al. 2019; Gungor et al. 2018; Martino et al. 2010; Peltier et al. 2006; Peuskens et al. 2004; Serra et al. 2017; Shah et al. 2012; Vergani et al. 2014; Verhaeghe et al. 2018; Wang et al. 2013; Wu et al. 2016a), or that their own modification (Burks et al. 2017; de Castro et al. 2005; Goryainov et al. 2017; Latini et al. 2015; Rigoard et al. 2011; Silva and Andrade 2016; Sincoff et al. 2004) of the Klingler’s technique is applied. This may be confusing for those who want to start their own dissections. Most commonly, the three main steps are the same in each laboratory, but the details of each vary between studies, sometimes significantly. The aim of this manuscript is to present our technique and review data about different techniques, which we hope will give readers a chance to choose the technique that best suits their needs.

## Methods

We conducted a literature review regarding the published literature relating the details of the different modifications of specimen preparation for white matter dissection with the Klingler technique. Ethics committee approval was obtained for the part of the study that presents the technique used in our laboratory. The approval number, AKBE/126/2019, was obtained from the Bioethics Committee of our University.

### Literature study

Three independent investigators performed an electronic literature search that was carried out in the Pubmed, Scopus and Web of Science databases up to December 2019. We used a [(Klingler) AND (Technique)] OR (White matter dissection) combination of keyword categories. Furthermore, we performed citation tracking for the articles missed through the initial search. The validity of the studies was assessed by the 3 investigators, and a consensus between them was used to resolve any disagreement. The database search identified 108 articles, and another 12 were found via citation tracking. Therefore, 120 full-text manuscripts were included in the final evaluation (Table 1). The methods sections of these 120 manuscripts were reviewed, and 37 articles eligible for inclusion in the study were identified (Table 2).

**Table 1:** List of all full-text manuscripts that were included for the evaluation

No	Title	First author	Year	Journal	Material	I step—formalin solution	I step—time	II step—temperature	II step—time	II step—preservation
1	Vertical bundles of the white matter fibers in the pons revisited: preliminary study utilizing the Klingler technique	Grzegorczyk M	2019	Anatomical Science International	Brain stem	10% formalin	N/A	− 20	24 h	N/A
2	Microsurgical anatomy of the sagittal stratum	Di Carlo D.T	2019	Acta Neurochirurgica	Brain	10% formalin	At least 4 weeks	− 15	15 days	N/A
3	White matter relationships examined by transillumination technique using a lateral transcortical parietal approach to the atrium: three-dimensional images and surgical considerations	Capilla-Guasch P	2019	World Neurosurgery	Brain and heads	10% formalin	At least 3 months	− 16	At least 14 days	5% formalin
4	Anatomy and white matter connections of the inferior frontal gyrus	Briggs R.G	2019	Clinical Anatomy	Brain	10% formalin	At least 3 months	− 10	8 h	N/A
5	Microsurgical anatomy of the insular region and operculoinsular association fibers and its neurosurgical application	Pastor-Escartín F	2019	World Neurosurgery	Brain	Formalin solution unknown	N/A	N/A	N/A	N/A
6	Neuroanatomical aspects of the temporo-parieto-occipital junction and new surgical strategy to preserve the associated tracts in junctional lesion surgery: fiber separation technique	Biceroglu H	2019	Turkish Neurosurgery	Brain and heads	Formalin solution unknown	N/A	− 16	At least15 days	N/A
7	Microsurgical anatomy of the vertical rami of the superior longitudinal fasciculus: an intraparietal sulcus dissection study	Monroy-Sosa A	2019	Operative Neurosurgery	Brain and heads	Formalin solution unknown	N/A	− 15	2 weeks	N/A
8	Inferior fronto-occipital fascicle anatomy in brain tumor surgeries: from anatomy lab to surgical theater	Altieri R	2019	Journal of Clinical Neurorscience	Brain	Formalin solution unknown	N/A	N/A	N/A	N/A
9	Real-time ex-vivo magnetic resonance imag—guided dissection of human brain white matter: a proof-of-principle study	Bertolini G	2019	World Neurosurgery	Brain	5% formalin	5 weeks	− 20	10 days	N/A
10	White matter topographic anatomy applied to temporal lobe surgery	Flores-Justa A	2019	World Neurosurgery	Head	10% formalin	30 days	− 15	15 days	N/A
11	Neural circuitry: architecture and function-a fiber dissection study	Shah A	2019	World Neurosurgery	Brain	10% formalin	30 days	− 10	3–4 weeks	4% formalin
12	Brainstem anatomy: a study on the basis of pattern of fiber organization	Shah A	2019	World Neurosurgery	Brain	10% formalin	30 days	− 10	4 weeks	4% formalin
13	Surgical approaches to the thalamus in relation to the white matter tracts of the cerebrum	Baran O	2019	World Neurosurgery	Brain and heads	10% formalin	3 weeks	− 16	At least 2 weeks	70% alcohol
14	Anatomy and white matter connections of the lateral occipital cortex	Palejwala A.H	2019	Surgical and Radiologic Anatomy	Brain	10% formalin	At least 3 months	− 10	8 h	N/A
15	The temporoinsular projection system: an anatomical study	Nachtergaele P	2019	Journal of Neurosurgery	Brain	10% formalin	At least 2 months	− 10	4 weeks	5% formalin
16	Structure, asymmetry, and connectivity of the human temporo-parietal aslant and vertical occipital fasciculi	Panesar S.S	2019	Brain Structure and Function	Brain	10% formalin	2 months	− 16	2 weeks	N/A
17	The frontal longitudinal system as revealed through the fiber microdissection technique: structural evidence underpinning the direct connectivity of the prefrontal-premotor circuitry	Komaitis S	2019	Journal of Neurosurgery	Brain	N/A	N/A	N/A	N/A	N/A
18	A white matter fiber microdissection study of the anterior perforated substance and the basal forebrain: a gateway to the basal ganglia	Serra C	2018	Operative Neurosurgery	Brain	Formalin solution unknown	N/A	N/A	N/A	N/A
19	Dorsal component of the superior longitudinal fasciculus revisited: novel insights from a focused fiber dissection study	Komaitis S	2019	Journal of Neurosurgery	Brain	N/A	N/A	N/A	N/A	N/A
20	Sledge runner fasciculus: anatomic architecture and tractographic morphology	Koutsarnakis C	2019	Brain Structure and Function	Brain	10–15% formalin	At least 8 weeks	N/A	N/A	N/A
21	A technical guide for fiber tract dissection of the internal capsule	Costa M	2018	Turkish Neurosurgery	Brain	10% formalin	from 2 to 3 months	From − 5 to − 10	from 7 to 14 days	N/A
22	Microsurgical anatomy of the subthalamic nucleus: correlating fiber dissection results with 3-T magnetic resonance imaging using neuronavigation	Gungor A	2018	Journal of Neurosurgery	Brain and heads	10% formalin	1 month	− 16	2 weeks	70% alcohol
23	The crossed frontal aslant tract: a possible pathway involved in the recovery of supplementary motor area syndrome	Baker C.M	2018	Brain and Behavior	Brain	10% formalin	At least 2 months	− 15	1 month	N/A
24	Topography of the human acoustic radiation as revealed by ex vivo fibers micro-dissection and in vivo diffusion-based tractography	Maffei C	2018	Brain Structure and Function	Brain	N/A	N/A	N/A	N/A	N/A
25	Prevention of postoperative visual field defect after the occipital transtentorial approach: anatomical study	Matsuo S	2018	Journal of Neurosurgery	Brain	N/A	N/A	N/A	N/A	N/A
26	Defining the relationship of the optic radiation to the roof and floor of the ventricular atrium: a focused microanatomical study	Koutsarnakis C	2018	Journal of Neurosurgery	Brain	10–15% formalin	8 weeks	N/A	N/A	N/A
27	Photogrammetry of the human brain: a novel method for 3D the quantitative exploration of the structural connectivity in neurosurgery and neurosciences	De Benedictis A	2018	World Neurosurgery	Brain	10% formalin	40 days	− 80	40 days	N/A
28	Endoscopic approach of the insula through the anterior middle temporal gyrus: a feasibility study in the laboratory	Corrivetti F	2017	Operative Neurosurgery	Brain	10% formalin	3 weeks	− 18	2 weeks	N/A
29	White matter connections of the inferior parietal lobule: a study of surgical anatomy	Burks JD	2018	Brain and Behavior	Brain	10% formalin	3 months	− 10	8 h	N/A
30	Posterior quadrant disconnection: a fiber dissection study	Verhaeghe A	2018	Operative Neurosurgery	Brain	10% formalin	2 months	− 10	4 weeks	N/A
31	Structure of corona radiata and tapetum fibers in ventricular surgery	Yakar F	2018	Journal of Clinical Neurorscience	Brain	5% formalin	2 months	− 10	10 days	N/A
32	3D microsurgical anatomy of the cortico-spinal tract and lemniscal pathway based on fiber micrfromissection and demonstration with tractography	Rodríguez-Mena R	2018	Neurocirugia	Brain and brain stem	10% formalin	2 months	From − 10 to − 15	7–10 days	5% formalin
33	Anatomy of the limbic white matter tracts as revealed by fiber dissection and tractography	Pascalau R	2018	World Neurosurgery	Brain	9% formalin	15 days	− 20	15 days	N/A
34	Endoscopic endonasal transclival approach to the ventral brainstem: anatomic study of the safe entry zones combining fiber dissection technique with 7 T magnetic resonance guided neuronavigation	Weiss A	2018	Operative Neurosurgery	Brain stems	N/A	N/A	N/A	N/A	N/A
35	Anatomy and white matter connections of the orbitofrontal gyrus	Burks J.D	2017	Journal of Neurosurgery	Brain	10% formalin	At least 3 months	− 10	8 h	N/A
36	Topographic classification of the thalamus surfaces related to microneurosurgery: a white matter fiber microdissection study	Serra C	2017	World Neurosurgery	Brain	10% formalin	At least 2 months	− 20	At least 7 days	N/A
37	Neurosurgical relevance of the dissection of the diencephalic white matter tracts using the Klingler technique	Silva S.M	2017	Clinical Neurology and Neurosurgery	Brain	10% formalin	At least 2 months	− 15	1 month	N/A
38	The white matter tracts of the cerebrum in ventricular surgery and hydrocephalus	Güngör A	2017	Journal of Neurosurgery	Brain and whole heads	Formalin solution unknown	N/A	− 16	2 weeks	70% alcohol
39	Microsurgical anatomy of the central core of the brain	Ribas E.C	2017	Journal of Neurosurgery	Brain	Formalin solution unknown + 70% alcohol	N/A	N/A	At least 1 month	N/A
40	Callosotopy: leg motor connections illustrated by fiber dissection	Naets W	2017	Brain Structure and Function	Brain	10% formalin	8 weeks	− 10	4 weeks	N/A
41	Approaching the atrium through the intraparietal sulcus: mapping the sulcal morphology and correlating the surgical corridor to underlying fiber tracts	Koutsarnakis C	2017	Operative Neurosurgery	Brain	10–15% formalin	6 weeks	N/A	N/A	N/A
42	Three-dimensional anatomy of the white matter fibers of the temporal lobe: surgical implications	Pescatori L	2017	World Neurosurgery	Brain	10% formalin	At least 40 days	− 15	14 days	N/A
43	Long association tracts of the human white matter: an analysis of 18 hemisphere dissections and in vivo HARDI-CSD tractography	Goryaynov S.A	2017	Zhurnal voprosy neirokhirurgii imeni N. N. Burdenko	Brain	10% formalin	4 weeks	− 20	1 week	96% alcohol
44	Neuronavigated fiber dissection with pial preservation: laboratory model to simulate opercular approaches to insular tumors	Mandonnet E	2017	World Neurosurgery	Brain	10% formalin	3 weeks	− 18	2 weeks	N/A
45	Fiber tracts of the medial and inferior surfaces of the cerebrum	Baydin S	2017	World Neurosurgery	Brain	10% formalin	3 weeks	− 16	N/A	70% alcohol
46	Segmentation of the inferior longitudinal fasciculus in the human brain: a white matter dissection and diffusion tensor tractography study	Latini F	2017	Brain Research	Whole body	12% formalin	24 h	From − 15 to − 20	From 6 to 10 days	N/A
47	Surgical approaches to the temporal horn: an anatomic analysis of white matter tract interruption	Kadri P.A.S	2017	Operative Neurosurgery	Brain	10% formalin	2 months	From − 10 to − 15	1 week	N/A
48	Fiber connections of the supplementary motor area revisited: methodology of fiber dissection, dti, and three dimensional documentation	Bozkurt B	2017	Journal of Visualized Experiments	Brain	10% formalin	2 months	− 16	2 weeks	N/A
49	The superior frontal transsulcal approach to the anterior ventricular system: exploring the sulcal and subcortical anatomy using anatomic dissections and diffusion tensor imaging tractography	Koutsarnakis C	2017	World Neurosurgery	Brain	10–15% formalin	2 months	N/A	N/A	N/A
50	Revisiting the human uncinate fasciculus, its subcomponents and asymmetries with stem-based tractography and microdissection validation	Hau J	2017	Brain Structure and Function	Brain	10% formalin	N/A	N/A	N/A	N/A
51	Neuroanatomy: the added value of the klingler method	Silva S.M	2016	Annals of Anatomy	Whole body	10% formalin	At least 4 weeks	− 15	1 month	10% formalin
52	Anatomic connections of the subgenual cingulate region	Vergani F	2016	Neurosurgery	Brain	10% formalin	At least 3 months	− 15	15 days	N/A
53	Fiber tracts of the dorsal language stream in the human brain	Yagmurlu K	2016	Journal of Neurosurgery	Brain and heads	N/A	N/A	− 16	2 weeks	N/A
54	Transcortical selective amygdalohippocampectomy technique through the middle temporal gyrus revisited: An anatomical study laboratory investigation	Bozkurt B	2016	Journal of Clinical Neurorscience	Whole heads	N/A	N/A	− 16	2 weeks	N/A
55	Microsurgical anatomy and internal architecture of the brainstem in 3d images: surgical considerations	Párraga R.G	2016	Journal of Neurosurgery	Brain	10% formalin	N/A	N/A	N/A	N/A
56	Microsurgical and fiber tract anatomy of the nucleus accumbens	Baydin S	2016	Operative Neurosurgery	Brain	Formalin solution unknown	N/A	− 16	2 weeks	70% alcohol
57	The cerebral isthmus: fiber tract anatomy, functional significance, and surgical considerations	Koutsarnakis C	2016	Journal of Neurosurgery	Brain and heads	10–15% formalin	6 weeks	N/A	N/A	N/A
58	Tracing short connections of the temporo-parieto-occipital region in the human brain using diffusion spectrum imaging and fiber dissection	Wu W	2016	Brain Research	Brain	10% formalin	40 days	− 16	15 days	N/A
59	Structural and functional integration between dorsal and ventral language streams as revealed by blunt dissection and direct electrical stimulation	Sarubbo S	2016	Human Brain Mapping	Brain	10% formalin	40 days	− 80	30 days	N/A
60	New insights in the homotopic and heterotopic connectivity of the frontal portion of the human corpus callosum revealed by microdissection and diffusion tractography	De Benedictis A	2016	Human Brain Mapping	Brain and heads	10% formalin	40 days	− 80	30 days	N/A
61	Segmentation of the cingulum bundle in the human brain: a new perspective based on dsi tractography and fiber dissection study	Wu Y	2016	Frontiers in Neuroanatomy	Brain	10% formalin	4 weeks	− 15	15 days	N/A
62	How klingler’s dissection permits exploration of brain structural connectivity? An electron microscopy study of human white matter	Zemmoura I	2016	Brain Structure and Function	Brain	5% formalin	3 months	− 23	3 weeks	N/A
63	Microsurgical and tractographic anatomy of the supplementary motor area complex in humans	Bozkurt B	2016	World Neurosurgery	Brain and heads	Formalin solution unknown	N/A	− 16	2 weeks	N/A
64	Microsurgical anatomy of the inferior limiting insular sulcus and the temporal stem	Ribas EC	2015	Journal of Neurosurgery	Brain	Formalin solution unknown	N/A	N/A	N/A	70% alcohol
65	A laboratory manual for stepwise cerebral white matter fiber dissection	Koutsarnakis C	2015	World Neurosurgery	Brain	10–15% formalin	6 weeks	From − 10 to − 15	15 days	N/A
66	The course and the anatomo-functional relationships of the optic radiation: a combined study with ‘post mortem’ dissections and ‘in vivo’ direct electrical mapping	Sarubbo S	2015	Journal of Anatomy	Brain	10% formalin	40 days	− 20	30 days	N/A
67	Subcortical anatomy as an anatomical and functional landmark in insulo-opercular gliomas: implications for surgical approach to the insular region	Martino J	2015	Journal of Neurosurgery	Brain	10% formalin	40 days	N/A	N/A	N/A
68	New Insights in the Limbic Modulation of Visual Inputs: The Role of the Inferior Longitudinal Fasciculus and the Li-Am Bundle	Latini F	2015	Neurosurgical Review	Brain	10% formalin	40 days	− 35	48 h	Alcohol solution
69	The controversial existence of the human superior fronto-occipital fasciculus: Connectome-based tractographic study with microdissection validation	Meola A	2015	Human Brain Mapping	Brain	10% formalin	4 weeks	− 16	2 weeks	N/A
70	Asymmetry, connectivity, and segmentation of the arcuate fascicle in the human brain	Fernandez-Miranda JC	2015	Brain Structure and Function	Brain	10% formalin	4 weeks	− 16	2 weeks	N/A
71	The nondecussating pathway of the dentatorubrothalamic tract in humans: human connectome-based tractographic study and microdissection validation	Meola A	2015	Journal of Neurosurgery	Brain	10% formalin	4 weeks	− 16	2 weeks	N/A
72	The use of a cerebral perfusion and immersion–fixation process for subsequent white matter dissection	Latini F	2015	Journal of Neuroscience Methods	Brain	10% formalin	24 h	From − 15 to − 20	from 6 to 10 days	5% formalin
73	Rethinking the standard trans-cortical approaches in the light of superficial white matter anatomy	Latini F	2015	Neural Regeneration Research	Brain	10% formalin	24 h	From − 15 to − 20	from 6 to 10 days	N/A
74	The classical pathways of occipital lobe epileptic propagation revised in the light of white matter dissection	Latini F	2015	Behavioural Neurology	Brain	10% formalin	24 h	From − 15 to − 20	from 6 to 10 days	5% formalin
75	The anatomy of Meyer's loop revisited: changing the anatomical paradigm of the temporal loop based on evidence from fiber micodissection	Goga C	2015	Journal of Neurosurgery	Brain	N/A	N/A	N/A	N/A	N/A
76	Preservation of the optic radiations based on comparative analysis of diffusion tensor imaging tractography and anatomical dissection	Nooij R.P	2015	Frontiers in Neuroanatomy	Brain	N/A	N/A	From − 15 to − 20	2 weeks	N/A
77	The dentate nucleus and its projection system in the human cerebellum: the dentate nucleus microsurgical anatomical study	Akakin A	2014	Neurosurgery	Brain (cerebellum)	N/A	N/A	N/A	N/A	N/A
78	Anatomic study of the central core of the cerebrum correlating 7-T magnetic resonance imaging and fiber dissection with the aid of a neuronavigation system	Alarcon C	2014	Operative Neurosurgery	Brain	10% formalin	N/A	N/A	N/A	N/A
79	Anatomo-functional study of the temporo-parieto-occipital region: dissection, tractographic and brain mapping evidence from a neurosurgical perspective	De Benedictis A	2014	Journal of Anatomy	Brain	10% formalin	40 days	− 20	30 days	N/A
80	FIBRASCAN: a novel method for 3D white matter tract reconstruction in MR space from cadaveric dissection	Zemmoura I	2014	Neuroimage	Brain	5% formalin	4 months	− 23	7 days	2,5% formalin
81	Intralobar fibres of the occipital lobe: a postmortem dissection study	Vergani F	2014	Cortex	Brain	10% formalin	3 months	− 15	15 days	N/A
82	White matter connections of the supplementary motor area in humans	Vergani F	2014	Journal of Neurology Neurosurgery and Psychiatry	Brain	10% formalin	3 months	− 15	15 days	N/A
83	Evidence of a middle longitudinal fasciculus in the human brain from fiber dissection	Maldonado I.L	2013	Journal of Anatomy	Brain	N/A	N/A	− 20	2–4 weeks	N/A
84	Fiber dissection and diffusion tensor imaging tractography study of the temporoparietal fiber intersection area	Martino J	2013	Neurosurgery	Brain	10% formalin	40 days	− 15	15 days	N/A
85	Frontal terminations for the inferior fronto-occipital fascicle: anatomical dissection, DTI study and functional considerations on a multi-component bundle	Sarubbo S	2013	Brain Structure and Function	Brain	10% formalin	40 days	− 15	15 days	N/A
86	Analysis of the subcomponents and cortical terminations of the perisylvian superior longitudinal fasciculus: a fiber dissection and DTI tractography study	Martino J	2013	Brain Structure and Function	Brain	10% formalin	40 days	− 15	15 days	N/A
87	Rethinking the role of the middle longitudinal fascicle in language and auditory pathways	Wang Y	2013	Cerebral Cortex	Brain	10% formalin	4 weeks	− 16	2 weeks	N/A
88	Reproducibility of quantitative fiber tracking measurements in diffusion tensor imaging of frontal lobe tracts: a protocol based on the fiber dissection technique	Dini L.I	2013	Surgical Neurology International	Brain	10% formalin	2 months	− 15	1 week	5% formalin
89	Technical, anatomical, and functional study after removal of a symptomatic cavernous angioma located in deep wernicke's territories with cortico-subcortical awake mapping	Sarubbo S	2013	Case Reports in Neurological Medicine	Brain	10% formalin	N/A	− 20	N/A	N/A
90	Dorsal fronto-parietal connections of the human brain: a fiber dissection study of their composition and anatomical relationships	Maldonado I.L	2012	The Anatomical Record	Brain	Formalin solution unknown	N/A	− 20	14 days	N/A
91	Subcortical surgical anatomy of the lateral frontal region: human white matter dissection and correlations with functional insights provided by intraoperative direct brain stimulation	De Benedictis A	2012	Journal of Neurosurgery	Brain	10% formalin	40 days	− 15	15 days	N/A
92	Microsurgical anatomy of the optic radiation and related fibers in 3-dimensional images	Párraga R.G	2012	Operative Neurosurgery	Brain	Formalin solution unknown	40 days	− 15	14 days	N/A
93	Analysis of the anatomy of the Papez circuit and adjoining limbic system by fiber dissection techniques	Shah A	2012	Journal of Clinical Neurorscience	Brain	10% formalin	30 days	− 10	3–4 weeks	4% formalin
94	Cortex-sparing fiber dissection: an improved method for the study of white matter anatomy in the human brain	Martino J	2011	Journal of Anatomy	Brain	10% formalin	40 days	− 15	15 days	4% formalin
95	Microsurgical anatomy of the posterior cerebral artery in three-dimensional images	Párraga R.G	2011	World Neurosurgery	Brain	10% formalin	40 days	From − 10 to − 15	14 days	N/A
96	Microsurgical anatomy of the anterior commissure: correlations with diffusion tensor imaging fiber tracking and clinical relevance	Peltier J	2011	Operative Neurosurgery	Brain	10% formalin	3 months	From − 10 to − 15	15 days	N/A
97	Temporal lobe association fiber tractography as compared to histology and dissection	Holl N	2011	Surgical and Radiologic Anatomy	Brain	N/A	N/A	− 20	8 days	4% formalin
98	Anatomic dissection of the inferior fronto-occipital fasciculus revisited in the lights of brain stimulation data	Martino J	2010	Cortex	Brain	10% formalin	40 days	− 15	15 days	N/A
99	New insights into the anatomic dissection of the temporal stem with special emphasis on the inferior fronto-occipital fasciculus: implications in surgical approach to left mesiotemporal and temporoinsular structures	Martino J	2010	Neurosurgery	Brain	10% formalin	40 days	− 15	15 days	N/A
100	White fiber dissection of brain; the internal capsule: a cadaveric study	Chowdhury F	2010	Turkish Neurosurgery	Brain	10% formalin	3–6 months	N/A	4 weeks	N/A
101	Microsurgical anatomy of the temporal stem: clinical relevance and correlations with diffusion tensor imaging fiber tracking	Peltier J	2010	Journal of Neurosurgery	Brain	10% formalin	3 months	From − 10 to − 15	15 days	N/A
102	The accumbofrontal fasciculus in the human brain: a microsurgical anatomical study	Philippe R	2010	Neurosurgery	Brain	10% formalin	24 h	− 18	24 h	N/A
103	Image-guided dissection of human white matter tracts as a new method of modern neuroanatomical training	Skadorwa T	2009	Folia Morphologica	Brain	4% formalin	N/A	− 10	N/A	N/A
104	Fiber dissection of the visual pathways: analysis of the relationship of optic radiations to lateral ventricle: a cadaveric study	Pujari V.B	2008	Neurology India	Brain	Formalin solution unknown	40 days	From − 10 to − 15	14 days	N/A
105	The claustrum and its projection system in the human brain: a microsurgical and tractographic anatomical study	Fernandez-Miranda J.C	2008	Journal of Neurosurgery	Brain	10% formalin	40 days	From − 10 to − 15	14 days	N/A
106	Three-dimensional Microsurgical and Tractographic Anatomy of the White Matter of the Human Brain	Fernandez-Miranda J.C	2008	Neurosurgery	Brain	10% formalin	3 weeks	− 16	2–4 weeks	N/A
107	Anatomic relationship of the optic radiations to the atrium of the lateral ventricle: description of a novel entry point to the trigone	Mahaney K.B	2008	Operative Neurosurgery	Brain	4% formalin	10 days	− 10	24 h	4% formalin
108	Meyer's loop and the optic radiations in the transsylvian approach to the mediobasal temporal lobe	Choi C	2006	Operative Neurosurgery	Brain	Formalin solution unknown	40 days	From − 10 to − 15	14 days	N/A
109	Optic radiations: a microsurgical anatomical study	Peltier J	2006	Journal of Neurosurgery	Brain	10% formalin	3 months	From − 10 to − 15	15 days	N/A
110	Three-dimensional Relationships of the Optic Radiation	Rubino P.A	2005	Neurosurgery	Brain	Formalin solution unknown	40 days	From − 10 to − 15	14 days	N/A
111	Internal structure of the cerebral hemispheres: an introduction of fiber dissection technique	De Castro I	2005	Arquivos de Neuro-Psiquiatria	Brain	10% formalin	4 weeks	− 10	8 days	10% formalin
112	An anteromedial approach to the temporal horn to avoid injury to the optic radiation fibers and uncinate fasciculus: anatomical and technical note	Coppens J.R	2005	Neurosurgical Focus	Brain	4% formalin	10 days	− 10	24 h	4% formalin
113	Anatomy of the anterior temporal lobe and the frontotemporal region demonstrated by fiber dissection	Peuskens D	2004	Neurosurgery	Brain	10% formalin	4 weeks	− 12	4 weeks	N/A
114	White matter fiber dissection of the optic radiations of the temporal lobe and implications for surgical approaches to the temporal horn	Sincoff E.H	2004	Journal of Neurosurgery	Brain	4% formalin	10 days	− 10	24 h	N/A
115	Fiber dissection technique: lateral aspect of the brain	Ture U	2000	Neurosurgery	Brain	10% formalin	At least 2 months	From − 10 to − 15	7 days	N/A
116	Is there a superior occipitofrontal fasciculus? a microsurgical anatomic study	Ture U	1997	Neurosurgery	Brain	10% formalin	At least 2 months	From − 10 to − 15	7 days	N/A
117	Subcortical topography and proportions of the pyramidal tract	Ebeling U	1992	Acta Neurochirurgica	Brain	Formalin solution unknown	2–4 weeks	From − 8 to − 10	"some days"	N/A
118	Neurosurgical topography of the optic radiation in the temporal lobe	Ebeling U	1988	Acta Neurochirurgica	Brain	Formalin solution unknown	2–4 weeks	From − 8 To − 10	"some days"	N/A
119	The connections of the amygdala and of the anterior temporal cortex in the human brain	Klingler J., Ludwig E	1960	Journal of Comparative Neurology	Brain	5% formalin	At least 2 months	From − 10 to − 15	from 8 to 10 days	5% formalin
120	The preparation method of klingler	Klingler J., Ludwig E	1956	Atlas Cerebri Humani	Brain	5% formalin	At least 4 weeks	From − 8 to − 10	8 days	5% formalin

**Table 2:** General characteristics of the studies and the main details about the technique used in each study

No	Title	First author	I step—Formalin solution	I step—Time	II step—Temperature	II step—Time	Preservation
0	Atlas cerebri humani	Ludwig E.; Klingler J	5% formalin	4 weeks	From − 8 to − 10	8 days	5% formalin
0	The connections of the amygdala and of the anterior temporal cortex in the human brain	Klingler J.; Gloor P	5% formalin	2–3 months	From − 10 to − 15	from 8 to 10 days	5% formalin
1	Neurosurgical laboratory in author's center	Dziedzic T	4% formalin	4 weeks	− 15	2 weeks	5% formalin
2	Fiber dissection technique: lateral aspect of the brain	Türe U	10% formalin	2 months	From − 10 to − 15	1 week	5% formalin
3	White matter fiber dissection of the optic radiations of the temporal lobe and implications for surgical approaches to the temporal horn	Sincoff E	4% formalin	10 days	− 10	24 h	4% formalin
4	Anatomy of the Anterior Temporal Lobe and the Frontotemporal Region Demonstrated by Fiber Dissection	Peuskens D	10% formalin	4 weeks	− 12	4 weeks	"lower formalin concetration"
5	Internal structure of the cerebral hemispheres: an introduction of fiber dissection technique	de Castro I	10% formalin	4 weeks	− 10	8 days	10% formalin
6	Optic radiations: a microsurgical anatomical study	Peltier J	10% formalin	3 months	From − 10 to − 15	15 days	N/A
7	The claustrum and its projection system in the human brain: a microsurgical and tractographic anatomical study	Fernandez-Miranda J	10% formalin	40 days	From − 10 to − 15	14 days	N/A
8	The Accumbofrontal Fasciculus in the Human Brain: A Microsurgical Anatomical Study	Rigoard P	10% formalin	24 h	− 18	24 h	4% formalin
9	Anatomic dissection of the inferior fronto-occipital fasciculus revisited in the lights of brain stimulation data	Martino J. C	10% formalin	40 days	− 15	15 days	N/A
10	Analysis of the anatomy of the Papez circuit and adjoining limbic system by fiber dissection techniques	Shah A	10% formalin	30 days	− 10	3–4 weeks	4% formalin
11	Reproducibility of quantitative fiber tracking measurements in diffusion tensor imaging of frontal lobe tracts: a protocol based on the fiber dissection technique	Dini L. I	10% formalin	2 months	− 15	1 week	5% formalin
12	Rethinking the role of the middle longitudinal fascicle in language and auditory pathways	Wang Y	10% formalin	4 weeks	− 16	2 weeks	N/A
13	Intralobar fibres of the occipital lobe: a post mortem dissection study	Vergani F	10% formalin	3 months	− 15	15 days	N/A
14	Anatomo-functional study of the temporo-parieto-occipital region: dissection, tractographic and brain mapping evidence from a neurosurgical perspective	De Benedictis A	10% formalin	40 days	− 20	30 days	N/A
15	The use of a cerebral perfusion and immersion–fixation process for subsequent white matter dissection	Latini F	10% formalin	24 h	From − 10 to − 15	from 6 to 10 days	5% formalin
16	New insights in the limbic modulation of visual inputs: the role of the inferior longitudinal fasciculus and the li-am bundle	Latini F	10% formalin	40 days	− 35	2 days	alcohol
17	Neuroanatomy: the added value of the klingler method	Silva S. M	10% formalin	1 month	− 15	4 weeks	10% formalin
18	Neuronavigated fiber dissection with pial preservation: laboratory model to simulate opercular approaches to insular tumors	Mandonnet E	10% formalin	3 weeks	− 18	2 weeks	N/A
19	Segmentation of the cingulum bundle in the human brain: a new perspective based on dsi tractography and fiber dissection study	Wu Y	10% formalin	4 weeks	− 15	15 days	N/A
20	Structural and functional integration between dorsal and ventral language streams as revealed by blunt dissection and direct electrical stimulation	Sarubbo S	10% formalin	40 days	− 80	30 days	N/A
21	Tracing short connections of the temporo-parieto-occipital region in the human brain using diffusion spectrum imaging and fiber dissection	Wu Y	10% formalin	40 days	− 16	15 days	N/A
22	A laboratory manual for stepwise cerebral white matter fiber dissection	Koutsarnakis C	10–15% formalin	6 weeks	From − 10 to − 15	15 days	N/A
23	Topographic classification of the thalamus surfaces related to microneurosurgery: a white matter fiber microdissection study	Serra C	10% formalin	8 weeks	− 20	7 days	N/A
24	Fiber connections of the supplementary motor area revisited: methodology of fiber dissection, dti, and three dimensional documentation	Bozkurt B	10% formalin	2 months	− 16	2 weeks	10% formalin
25	Surgical approaches to the temporal horn: an anatomic analysis of white matter tract interruption	Kadri P. A. S	10% formalin	2 months	From − 10 to − 15	1 week	N/A
26	Neurosurgical relevance of the dissection of the diencephalic white matter tracts using the Klingler technique	Silva S. M	10% formalin	2 months	− 15	4 weeks	10% formalin
27	White matter connections of the inferior parietal lobule: A study of surgical anatomy	Burks J. D	10% formalin	3 months	− 10	8 h	N/A
28	Long association tracts of the human white matter: an analysis of 18 hemisphere dissections and in vivo HARDI-CSD tractography	Goryainov S. A	10% formalin	4 weeks	− 20	7 days	96% alcohol or 5% formalin
29	Three-Dimensional Anatomy of the White Matter Fibers of the Temporal Lobe: Surgical Implications	Pescatori L	10% formalin	40 days	− 15	14 days	N/A
30	Microsurgical anatomy of the subthalamic nucleus: correlating fiber dissection results with 3-T magnetic resonance imaging using neuronavigation	Gungor A	10% formalin	1 month	− 16	2 weeks	70% alcohol
31	Structure, asymmetry, and connectivity of the human temporo-parietal aslant and vertical occipital fasciculi	Panesar S. S	10% formalin	2 months	− 16	2 weeks	N/A
32	Posterior quadrant disconnection: a fiber dissection study	Verhaeghe A	10% formalin	2 months	− 10	4 weeks	N/A
33	Structure of corona radiata and tapetum fibers in ventricular surgery	Yakar F	5% formalin	2 months	− 10	10 days	N/A
34	A technical guide for fiber tract dissection of the internal capsule	Costa M	10% formalin	2–3 months	From − 5 to − 10	1–2 weeks	N/A
35	Microsurgical anatomy of the sagittal stratum	Di Carlo D. T	10% formalin	1 month	− 15	15 days	N/A
36	White matter topographic anatomy applied to temporal lobe surgery	Flores-Justa A	10% formalin	1 month	− 15	15 days	N/A
37	White matter relationships examined by transillumination technique using a lateral transcortical parietal approach to the atrium: three-dimensional images and surgical considerations	Capilla-Guasch P	10% formalin	3 months	− 16	14 days	5% formalin
38	Surgical approaches to the thalamus in relation to the white matter tracts of the cerebrum	Barand O	10% formalin	3 weeks	− 16	2 weeks	70% alcohol

### Study selection

We included original anatomical studies in which human white matter dissection was performed for research purposes. Studies were eligible for inclusion when they reported details of at least the first 2 main steps of Klingler’s technique: fixation and freezing. We limited our review to studies published in English. When multiple publications from the same laboratory were available and the same technique was described, we used the initial manuscript as a reference describing the technique in this review.

### Data extraction

Using a standardized data extraction form, 3 investigators independently extracted the following aspects from the studies included in the review: the number of hemispheres used for the study, type of fixing fluid, concentration of the solution used for fixation, time of fixation, temperature and duration of freezing, and the thawing technique. Additional data about the technique used to avoid brain disfigurement, the type of fluid and its concentration used between dissections, and the magnification technique and type of instruments used for dissection were also extracted from the studies. From all papers dealing with this topic, other features and exceptions from the main technique were also presented in the discussion.

### Our technique

**Fig. 1:**
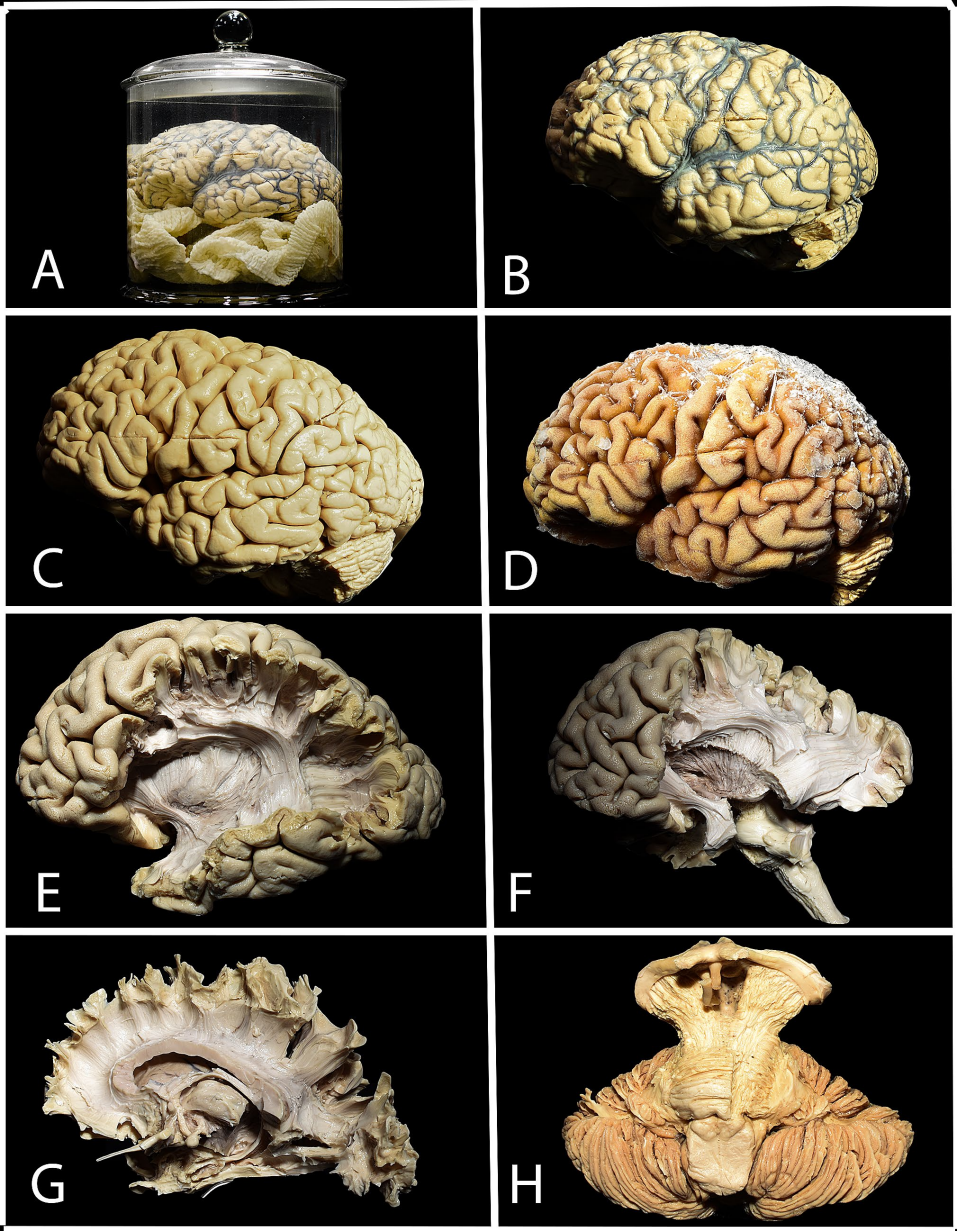
The preparation technique used in our laboratory. **a** The brain is placed within a container filled with 4% formalin and gauze to prevent deformation during the fixation process. **b**, **c** After washing out the formalin, the arachnoid and vessels are removed. **d** The dry brain is placed on the support inside the freezer with the temperature set at − 15 °C and kept there, usually, for 2 weeks. **e**–**h** Dissection is performed mainly with microsurgical instruments and under microsurgical microscope magnification

The brain is removed from the body on the next working day, except weekends where delay make take up to 72 h. The brain is placed within a container filled with 4% formalin and gauze to protect it from deformation during the fixation process (Fig. 1a). The brain is kept in this solution for at least 4 weeks. After washing out the formalin, the arachnoid and vessels are removed (Fig. 1b, c). The dry brain is placed on the support inside the freezer with the temperature set at − 15 °C and kept there for two weeks (Fig. 1d). For thawing, the specimen is placed in a container with 4% formalin solution at room temperature. Dissection is performed mainly with microsurgical instruments and under microsurgical microscope magnification (Fig. 1e–h). Two- and three-dimensional picture documentation is performed with a digital camera.

## Results

Details of the technique, including fixation, freezing and thawing, are described in detail in Table 2, which summarizes our results (Baran et al. 2019; Bozkurt et al. 2017; Burks et al. 2017; Capilla-Guasch et al. 2019; Costa et al. 2018; De Benedictis et al. 2014; de Castro et al. 2005; Di Carlo et al. 2019; Dini et al. 2013; Fernandez-Miranda et al. 2008; Flores-Justa et al. 2019; Goryainov et al. 2017; Gungor et al. 2018; Ludwig and Klingler 1956; Kadri et al. 2017; Koutsarnakis et al. 2015; Latini 2015; Latini et al. 2015; Mandonnet et al. 2017; Martino et al. 2010; Panesar et al. 2019; Pescatori et al. 2017; Peuskens et al. 2004; Rigoard et al. 2011; Sarubbo et al. 2016; Serra et al. 2017; Shah et al. 2012; Silva and Andrade 2016; Silva et al. 2017; Sincoff et al. 2004; Ture et al. 2000; Vergani et al. 2014; Verhaeghe et al. 2018; Wang et al. 2013; Wu et al. 2016a, b; Yakar et al. 2018).

### Fixation

In 6 out of 37 manuscripts, the authors mentioned the time between death, brain removal from the skull and initiation of the fixation process. The authors advised performing this process within the first 12 h in 4 manuscripts, within 8 h in 1 manuscript and in less than 18 h in 1 manuscript. For fixation, specimens were placed in formalin solution in all reviewed manuscripts, but the concentration of solution varied between the studies. Ten percent formalin solution was the most commonly used (34 studies). In 1 study, the concentration was higher and varied between 10 and 15% without mentioning a specific value. In 2 described techniques, the concentration of the solution was lower than 10% with values of 4–5%. The time of fixation varied from 24 h to 3 months. In 2 manuscripts, formalin solution was exchanged during the fixation process after the first 24 h and after 2 weeks. Most of the studies agreed that longer fixation is beneficial rather than harmful. To avoid brain deformity during the fixation process, the brain can be placed on gauze within the formalin solution and float freely (2 manuscripts), or the same effect can be achieved by placing a ligature on the basilar artery and using it to suspend the brain on wooden sticks placed at the top of the container (6 manuscripts). For anatomical studies, the authors used 2–60 hemispheres, with an average of 15 hemispheres per study.

### Freezing

In 17 manuscripts, an additional process of washing out formalin was mentioned between the fixation and freezing steps. It was performed with still or running water. This process takes from “several hours” up to 2 days. Twenty of 28 manuscripts reported that the arachnoid and vessels were removed before the freezing process had begun. In 7 of the remaining 8 manuscripts, it was done after freezing and in 1 manuscript, this process was performed between freezing-defreezing episodes. Multiple episodes (from 2 up to 5) of freezing-defreezing were performed as a standard procedure in 3 laboratories. The freezing time varied between 8 h and 1 month. The temperature at which the brain was preserved varied from − 5 to − 80 °C.

### Thawing and dissection

Thawing can be completed at room temperature on a tray or the specimen can be placed under running or in still water. After thawing and during dissections, the specimen was kept in formalin (13 manuscripts) or alcohol solution (4 manuscripts). The formalin solution concentration varied from 4 to 10%. In 1 manuscript, the authors stated that the concentration was lower than that used for fixation, which was 10%. In 9 manuscripts, it was lower than 10%. The alcohol solution concentration varied between 70% (2 manuscripts) and 96% (1 manuscript); in 1 manuscript, the author did not mention the concentration of the solution. In 1 manuscript, researchers used both alcohol and formalin but did not mention if there was any subjective difference between these two solutions. In 26 papers, it was mentioned that operative microscopy was beneficial for dissections. In another 2 manuscripts, the authors found the use of loops to be helpful. Thirty-one authors reported the tools that they used for dissection. Eleven used purely homemade wooden spatulas of different sizes, 11 used microsurgical instruments in addition to wooden spatulas, and the rest (9) used purely microsurgical dissectors.

## Discussion

White matter dissection was found to be useful not only for anatomical studies but also for simulating neurosurgical approaches to deep-seated intraaxial lesions (Mandonnet et al. 2017). The most commonly adopted brain preparation technique is one described by Klingler in his two manuscripts (Ludwig and Klingler 1956; Klingler and Gloor 1960).

In the first step of the preparation, the brain has to be removed from the skull, but some authors found it useful for anatomical studies to preserve the whole head (Baran et al. 2019; Bozkurt et al. 2017). This allows us to perfuse the cadaver vasculature with a silicon solution to better visualize brain arteries and veins. This also provides an opportunity to study the relationship between craniometric points and cortical and subcortical anatomical points. According to Klingler’s initial description, the brain should be removed from the body within 12 h of death. In 5 manuscripts that were chosen for review, the authors mentioned this step, and the time varied from less than 8–18 h (Di Carlo et al. 2019; Goryainov et al. 2017; Peltier et al. 2006; Rigoard et al. 2011; Ture et al. 2000). The rest of the authors did not mention this part of the preparation, which, as in our case, can be limited due to logistics issues. This part may not be very important as, according to Klingler, the blood content inside the brain is of greater importance than the time between death and removing the brain from the body. Greater amounts of blood inside the skull provide better differentiation between gray and white matter. This can be achieved by placing the head as the lowest point of the body. In none of the reviewed manuscript did the authors mention the importance of proper head positioning before removal of the brain from the skull. With standard techniques that are used during pathomorphological sections, it is easy to damage the lateral surface of the brain (Fig. 1b–d) (de Castro et al. 2005). Silva et al. mentioned the importance of careful removal of the calvaria and described in detail their technique to avoid damage to the underlying dura and the brain (Silva and Andrade 2016). Some authors prefer to start fixation before the brain is removed from the skull, and this is performed with formalin solution, which is injected intraarterially (Latini et al. 2015) (Silva and Andrade 2016; Silva et al. 2017), (Flores-Justa et al. 2019).

Klingler suggested that the brain should be suspended in fixing fluid, which is 5% formalin solution. All authors use formalin for fixation, but various solution concentrations are reported and vary from 4 to 15% (Sincoff et al. 2004; Wu et al. 2016b). Klingler used a concentration of 5% because he believed that a higher concentration of 10%, which is currently the most commonly used concentration, carries the risk of fixing the superficial parts of the hemisphere, yet the penetration of formalin at the 5% concentration to the deeper parts would not be sufficient. This may make the dissection more difficult, often unsatisfactory, and in some cases, even impossible to perform.

When the brain is removed from the body, it has a jelly-like consistency and is vulnerable to deformation. To preserve its shape during fixation, a wooden stick is placed at the top of the container, and the brain is suspended on a ligature placed on the basilar artery, which allows the brain to float freely in formalin solution (Costa et al. 2018; de Castro et al. 2005; Ludwig and Klingler 1956; Kadri et al. 2017; Silva and Andrade 2016; Ture et al. 2000). Other groups achieve the same effect by placing gauze inside the container, enabling free floating, so that the brain does not touch the sides of the container (Goryainov et al. 2017; Peuskens et al. 2004) (Fig. 1a). In the original description and in 2 other studies, the authors recommend exchanging the formalin solution after 24 h and 2 weeks (de Castro et al. 2005; Silva and Andrade 2016). The aim of this part of the preparation was not clarified in the reviewed studies. In total, the fixation process takes 4 weeks, but a longer period is beneficial according to Klingler and most authors (Ludwig and Klingler 1956). In the second manuscript about this technique, Klingler changed his technique and advocated that the brain should be kept in formalin solution for at least 2–3 months (Klingler and Gloor 1960). The reason he changed his practice was not mentioned. According to our review, satisfactory fixation can be achieved even after 24 h, but this may have a negative impact on the quality of the specimen and the ease of dissection (Rigoard et al. 2011).

Before the second step begins, Klingler recommended washing out the formalin from the brain under running water, which should take “several hours” (Kadri et al. 2017; Koutsarnakis et al. 2015; Silva and Andrade 2016; Ture et al. 2000; Wu et al. 2016a). The aim of this step is to remove the formalin solution, which is irritating to the observer during the next step, which includes removing the arachnoid and vessels (Baran et al. 2019; Capilla-Guasch et al. 2019; Costa et al. 2018; Di Carlo et al. 2019; Goryainov et al. 2017; Kadri et al. 2017; Koutsarnakis et al. 2015; Latini 2015; Latini et al. 2015; Martino et al. 2010; Panesar et al. 2019; Pescatori et al. 2017; Serra et al. 2017; Silva and Andrade 2016; Silva et al. 2017; Vergani et al. 2014; Wu et al. 2016a, b). Most authors removed the arachnoid before freezing except for a few who did it after this process is done or between episodes (Burks et al. 2017; De Benedictis et al. 2014; Gungor et al. 2018; Peuskens et al. 2004; Rigoard et al. 2011; Sarubbo et al. 2016; Shah et al. 2012; Sincoff et al. 2004). Adequately washing out formalin can be satisfactorily achieved with the brain placed in a container of still water (de Castro et al. 2005). In the next step, the brain is placed on a flat tray in a freezer for 8 days at a temperature of − 8 to − 10 °C. According to Klingler’s second manuscript, the temperature should be set between − 10° and − 15°, although the author did not define a specific temperature value. Lowering the temperature was combined with extending the freezing time for 2 days, which extended the overall freezing time from 8 to 10 days (Klingler and Gloor 1960). Some authors suggest that the freezing process can be performed when the brain is still in formalin, water or the alcohol solution (de Castro et al. 2005), (Goryainov et al. 2017). In the reviewed manuscripts, the temperature for the freezing step varied from − 5° to − 80° (Costa et al. 2018; Sarubbo et al. 2016). The time of freezing varied from 8 h to 8 weeks and did not correlate with the temperature. The aim of freezing is to spread myelinated nerve fibers apart, as the water solution increases 10% in volume with the formation of ice crystals. The crystals are placed between myelinated fibers as formalin solution does not penetrate the myelin sheath. This concept was proven recently with observations based on electron microscopy (Zemmoura et al. 2016). In 3 laboratories, the freezing–thawing technique is used, which is believed to provide better penetration of formalin solution between myelinated fibers (de Castro et al. 2005; Rigoard et al. 2011; Sincoff et al. 2004). After that time, the brain is thawed under running water (Bozkurt et al. 2017; de Castro et al. 2005; Gungor et al. 2018; Ludwig and Klingler 1956; Martino et al. 2010; Peuskens et al. 2004; Silva and Andrade 2016; Silva et al. 2017) or still water (Dini et al. 2013; Klingler and Gloor 1960; Serra et al. 2017; Shah et al. 2012; Ture et al. 2000) or is placed within formalin solution. This ends the preparation and makes the brain ready for dissection.

According to the initial description, the brain can be kept in 5% formalin solution, but for overnight or longer periods, the specimen should be kept in 2% solution. To minimize the unpleasant formalin odor, the brain in most of the manuscripts is kept in solution with lower formalin concentrations than those used for fixation (Capilla-Guasch et al. 2019; Costa et al. 2018) or in alcohol (Baran et al. 2019; Goryainov et al. 2017; Gungor et al. 2018; Peuskens et al. 2004). Some authors recommend re-freezing for a short period, such as 12 h, when there has to be a longer period without dissection (Goryainov et al. 2017; Ture et al. 2000). Refreezing is also useful for obtaining better definition when a dissection reaches deeper layers of white matter (Sarubbo et al. 2016).

Instruments initially used for dissection were Swiss watchmaker forceps and wooden spatulas, which are 2–4 mm wide, while metal spatulas were not recommended (Ludwig and Klingler 1956; Klingler and Gloor 1960). Most laboratories currently use wooden spatulas (Capilla-Guasch et al. 2019; Costa et al. 2018; De Benedictis et al. 2014; de Castro et al. 2005; Di Carlo et al. 2019; Dini et al. 2013; Flores-Justa et al. 2019; Latini et al. 2015; Panesar et al. 2019; Pescatori et al. 2017; Sarubbo et al. 2016; Serra et al. 2017; Shah et al. 2012; Silva and Andrade 2016; Sincoff et al. 2004; Ture et al. 2000; Vergani et al. 2014), but microsurgical metallic instruments (Capilla-Guasch et al. 2019; Costa et al. 2018; de Castro et al. 2005; Flores-Justa et al. 2019; Gungor et al. 2018; Koutsarnakis et al. 2015; Latini 2015; Latini et al. 2015; Martino et al. 2010; Peltier et al. 2006; Peuskens et al. 2004; Serra et al. 2017; Silva and Andrade 2016; Vergani et al. 2014; Wang et al. 2013; Wu et al. 2016b) have also been found to be useful for precise dissections. For a delicate dissection of low volume tracts, Klingler used snipe feathers, wet hair pencils or wet pieces of cotton wool (Klingler and Gloor 1960).

Most dissections are performed with the unaided eye, which is sufficient when the main cerebral tracts are dissected. For a better visualization of loops(Latini 2015; Martino et al. 2010), a microsurgical microscope can provide higher magnification and illumination (Baran et al. 2019; Bozkurt et al. 2017; de Castro et al. 2005; Fernandez-Miranda et al. 2008; Flores-Justa et al. 2019; Gungor et al. 2018; Koutsarnakis et al. 2015; Latini et al. 2015; Peltier et al. 2006; Peuskens et al. 2004; Rigoard et al. 2011; Serra et al. 2017; Shah et al. 2012; Ture et al. 2000; Verhaeghe et al. 2018; Wang et al. 2013; Wu et al. 2016a, b). In the third step, dissection starts with the removal of the whole hemisphere cortex to expose “U” fibers (Peuskens et al. 2004). This technique can later be confusing due to the loss of cortical anatomical landmarks. Therefore, Martino et al. recently presented a new technique in which the superficial cerebral cortex is preserved and dissection starts at the depth of the sulcus (Martino et al. 2011).

To document the results of dissection, Klingler used an analog camera with four-lamp illumination. Recently, this process has been performed with digital cameras that can potentially improve the quality of images with software dedicated for postprocessing (Bozkurt et al. 2017; Capilla-Guasch et al. 2019; Flores-Justa et al. 2019; Gungor et al. 2018; Kadri et al. 2017; Latini 2015; Martino et al. 2010; Pescatori et al. 2017; Sarubbo et al. 2016; Shah et al. 2012; Vergani et al. 2014; Wu et al. 2016b). Some authors believe that only photographs without any postprocessing are able to present realistic dissection results (Koutsarnakis et al. 2015). The same authors also suggest that taking pictures without the flash may be superficial in terms of fiber tract delineation (Koutsarnakis et al. 2015). Two- or three-dimensional pictures can be taken (Baran et al. 2019; Bozkurt et al. 2017; Capilla-Guasch et al. 2019).

According to our review, some authors changed their preparation technique, but there is lack of information regarding what made them do so and if there was any benefit from a dissection quality point of view (Silva and Andrade 2016; Silva et al. 2017).

For the main white matter tracts high replicability of Klingler methods is observed between the studies. Despite different techniques, the main tracts are easily identifiable, but the ease of dissection is objectively difficult to be assessed. Despite the same anatomical localization of the tracts different definitions across publications lead to the differences in the terminology. Anatomical background about white matter anatomy before performing dissection is mandatory. For better anatomical orientation, it is possible to perform dissections with the aid of MRI-based neuronavigation and custom-made fiducial markers (Skadorwa et al. 2009).

The limitation of this technique, except for being time-consuming and requiring great anatomical knowledge and manual skills, is that it requires good quality specimens and proper technique preparation. This was not the focus of our study, but in the majority of the manuscripts, our subjective assessment of the quality of the dissection based on the available figures was very high.

None of the studies compared different preservation techniques in terms of the quality of dissections or tract visualization in a subjective or objective way.

## Conclusion

There is an agreement between studies that the Klingler technique has three main steps: fixing, freezing and thawing with dissection. Even though the details of the technique are different in most of the studies, all provide good quality specimens for anatomical dissections and anatomical studies. This shows that those who wish to start this kind of dissection should choose the technique that fits them the best and follow it. Possible goals for other studies should include a comparison between different techniques in terms of the visualization of tracts and the ease of dissection.
